# A WD40 Repeat Protein from *Camellia sinensis* Regulates Anthocyanin and Proanthocyanidin Accumulation through the Formation of MYB–bHLH–WD40 Ternary Complexes

**DOI:** 10.3390/ijms19061686

**Published:** 2018-06-06

**Authors:** Yajun Liu, Hua Hou, Xiaolan Jiang, Peiqiang Wang, Xinlong Dai, Wei Chen, Liping Gao, Tao Xia

**Affiliations:** 1 School of Life Science, Anhui Agricultural University, Hefei 230036, China; liuyajun1228@163.com (Y.L.); houhua2015@163.com (H.H.); 2 State Key Laboratory of Tea Plant Biology and Utilization, Anhui Agricultural University, Hefei 230036, China; jiangxiaolan@ahau.edu.cn (X.J.); wpqtea@163.com (P.W.); xinlongdai@163.com (X.D.); chenwei2551@163.com (W.C.)

**Keywords:** *Camellia sinensis*, anthocyanin biosynthesis, CsWD40, flavan-3-ols, MYB–bHLH–WD40 complex

## Abstract

Flavan-3-ols and oligomeric proanthocyanidins (PAs) are the main nutritional polyphenols in green tea (*Camellia sinensis*), which provide numerous benefits to human health. To date, the regulatory mechanism of flavan-3-ol biosynthesis in green tea remains open to study. Herein, we report the characterization of a *C. sinensis* tryptophan-aspartic acid repeat protein (CsWD40) that interacts with myeloblastosis (MYB) and basic helix-loop-helix (bHLH) transcription factors (TFs) to regulate the biosynthesis of flavan-3-ols. Full length *CsWD40* cDNA was cloned from leaves and was deduced to encode 342 amino acids. An in vitro yeast two-hybrid assay demonstrated that CsWD40 interacted with two bHLH TFs (CsGL3 and CsTT8) and two MYB TFs (CsAN2 and CsMYB5e). The overexpression of *CsWD40* in *Arabidopsis thaliana transparent testa glabra 1* (*ttg1*) restored normal trichome and seed coat development. Ectopic expression of *CsWD40* alone in tobacco resulted in a significant increase in the anthocyanins of transgenic petals. *CsWD40* was then coexpressed with *CsMYB5e* in tobacco plants to increase levels of both anthocyanins and PAs. Furthermore, gene expression analysis revealed that *CsWD40* expression in tea plants could be induced by several abiotic stresses. Taken together, these data provide solid evidence that CsWD40 partners with bHLH and MYB TFs to form ternary WBM complexes to regulate anthocyanin, PA biosynthesis, and trichome development.

## 1. Introduction

Flavonoids are widely distributed and ubiquitous secondary metabolites in the plant kingdom [1]. Flavonoid compounds have been confirmed to be involved in various important physiological functions in plants, such as seed germination, protection from ultraviolet radiation, and pathogenic microorganism defense erosion [2,3,4]. More importantly, flavonoids have health benefits to human beings, such as antioxidative, antihypertensive, anti-inflammatory, antiaging, and insulin-sensitizing activities [5,6,7].

In plants, chalcones, flavones, flavonols, flavan-3-ols, anthocyanins, and proanthocyanidins (PAs, also called condensed tannins) are the primary subgroups of flavonoids [1]. Their biosynthetic pathways have been extensively studied over the past three decades [1,8,9]. The main pathway enzymes involved in flavonoid biosynthesis include chalcone synthase (CHS), chalconeisomerase (CHI), flavanone 3-hydroxylase (F3H), flavonoid 3′-hydroxylase (F3′H), flavonoid 3′,5′-hydroxylase (F3′5′H), flavonol synthase (FLS), dihydroflavonol 4-reductase (DFR), anthocyanidin synthase (ANS), and anthocyanidin reductase (ANR) [1].

In addition, regulation of the flavonoid pathway has been studied in-depth. To date, many transcription factors (TFs) have been identified from different plant species. Three TF families, WD40, basic helix-loop-helix, and MYB (v-myb avian myeloblastosis viral oncogene homolog), have been extensively studied to elucidate the regulation mechanism.

Numerous past studies have demonstrated that these three families form MBW (MYB-bHLH-WD40) ternary complexes to regulate the biosynthesis of anthocyanins and PA in all investigated plants, and the development of root hairs and trichomes in some plants [10,11,12,13,14]. For example, in *Arabidopsis*, four MBW complexes, PAP1(MYB75)–TT8/GL3–TTG1(WD40), PAP2(MYB90)–TT8/GL3–TTG1, MYB113(PAP3)–TT8/GL3–TTG1, and PAP4(MYB114)–TT8/GL3–TTG1 have been shown to activate the expression of late anthocyanin biosynthetic genes, such as *AtDFR*, *AtANS/LDOX*, and *UF3GT* [10,15,16]. The MBW complex TT2 (MYB123)–TT8/GL3–TTG1 regulated the expression of *AtDFR*, *AtANS/LDOX*, *AtANR/BANYULS* (*BAN*), glutathione-*S*-transferase (*TT19*), and a MATE (multidrug and toxic compound extrusion) transporter-encoding (*TT12*) involved in the PA biosynthesis [16,17].

In all of the MBW complexes, the WD40 repeat protein is localized in the center of ternary structure [18,19]. Unlike the bHLH and MYB members, WD40 has been shown to functionally enhance complex activation rather than directly participate in the recognition of the target gene promoter [20]. To date, several other WD40 repeat proteins that are orthologs to *Arabidopsis TTG1* have been reported in a few of plant species, such as *Perilla frutescens*, petunia (*Petunia hybrida*), cotton (*Gossypium hirsutum*), and maize (*Zea mays*). It is interesting that the gene encoding WD40 has been shown to have only one copy in the same species [21,22,23].

To date, the biosynthetic pathways of flavan-3-ols and PAs have gained intensive studies for human health benefits. Recently, the genome of green tea has been sequenced from different cultivars. These studies provide important knowledge to enhance tea industries for improved health benefits. However, the regulation mechanism of the flavonoid pathway in green tea remains to be elucidated. Herein, we report the cloning and functional analysis of cDNA encoding a WD40 repeat protein from green tea, namely *CsWD40*. Transgenic analysis, genetic complementation, and protein–protein interactions were performed to characterize that CsWD40 partner with R2R3-MYB and bHLH to form complexes which regulate the biosynthesis of anthocyanins, flavan-3-ols, and PAs. All data are fundamental to comprehensively elucidate the regulation of the green tea flavonoid pathway.

## 2. Results

### 2.1. Molecular Cloning and Sequence Analysis of CsWD40

The full-length cDNA of *CsWD40* was obtained from tender leaves of the local cultivar *C. sinensis Nongkangzao*’ through rapid amplification of cDNA ends polymerase chain reaction (RACE-PCR). The 1029-bp open reading frame (ORF) of *CsWD40* codes a protein with 342 amino acid residues. Alignment with CsWD40 proteins from other species revealed that four WD40 repeat domains are highly conserved among all WD40 repeat proteins (Figure S1). *CsWD40* shared 79.62% and 77.17% identity with *MdTTG1* (GU173813) from *Malus domestica* and *PgTTG1* (HQ199314) from *Punicagranatum*, respectively (Figure S1). *MdTTG1* and *PgTTG1* TFs have been reported to regulate anthocyanin biosynthesis in *M. domestica* and *P. granatum* plants, respectively [24,25]. Therefore, their orthologous gene *CsWD40* was predicted to be a TF that regulates anthocyanin biosynthesis in tea plants.

### 2.2. CsWD40 Interacts with MYB and bHLH TFs

The bHLH type TF genes *CsTT8* and *CsGL3* in *C. sinensis* are the orthologs of *AtTT8* and *AtGL3* in *Arabidopsis thaliana*, respectively. The MYB type TF genes *CsAN2* and *CsMYB5e* are the orthologs of *AtMYB75* in *A. thaliana* and *MtMYB5* in *M. truncatula*, respectively. *At*TT8, *At*GL3, and *At*TT2 have been demonstrated to form MBW complexes with WD40 proteins; these complexes regulate the phenylpropanoid pathway [20]. Therefore, CsWD40, CsMYB5e, CsTT8, and CsGL3 were selected to determine whether interactions occur among them in the yeast two-hybrid system.

**Figure 1:**
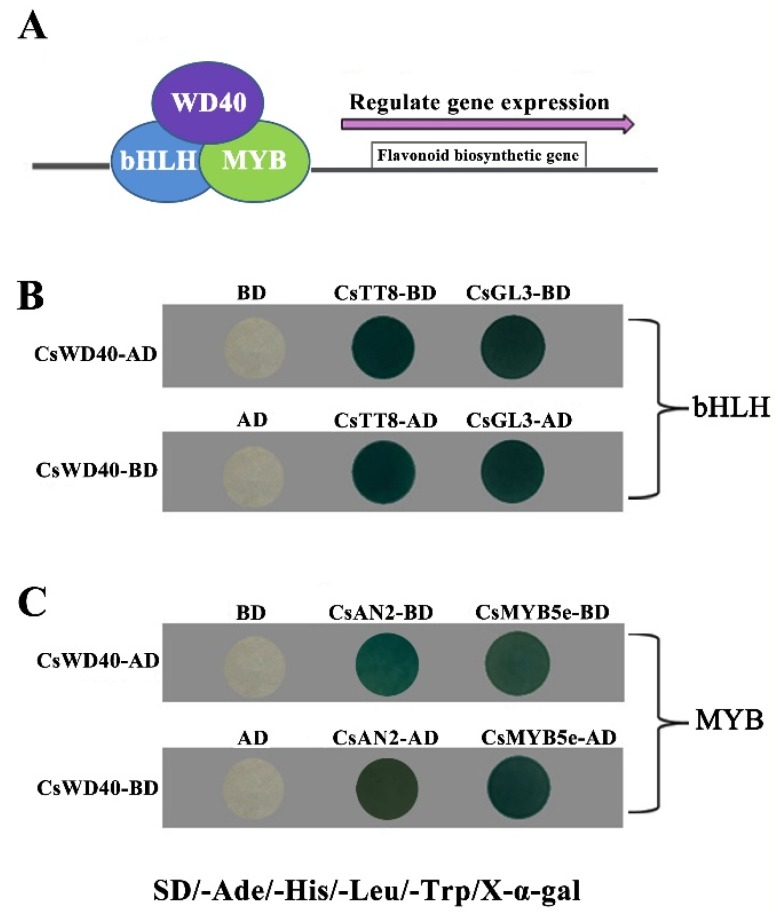
Protein interactions between CsWD40 and other transcript factors from tea plants by two-hybrid system. (**A**) A model pattern of the MYB–bHLH–WD40 ternary complex. (**B**) Protein–protein interactions between CsWD40 and bHLH transcript factors (CsTT8 and CsGL3) from tea plants by two-hybrid system. (**C**) Protein–protein interactions between *Cs*WD40 and MYB transcript factors (CsAN2 and CsMYB5e) from tea plants by two-hybrid system.

The yeast two-hybrid test results revealed that CsWD40 specifically interacted with the bHLH TFs CsTT8 and CsGL3 (Figure 1B). Moreover, both CsAN2 and CsMYB5e could physically interact with CsWD40 (Figure 1C). CsWD40 exhibited no autoactivation. The results indicated that CsGL3, CsTT8, CsAN2, CsMYB5e, and CsWD40 may be involved in jointly regulating the flavonoid pathway in tea plants.

### 2.3. CsWD40 Complements the Arabidopsis ttg1 Deficient Phenotype

In *A. thaliana*, *TTG1* not only regulates anthocyanin synthesis but is also involved in trichome organogenesis, seed coat pigment synthesis, root hair development, and regulation and control of negative cotyledon hypocotyl stomatal cell movement [18,19].

**Figure 2:**
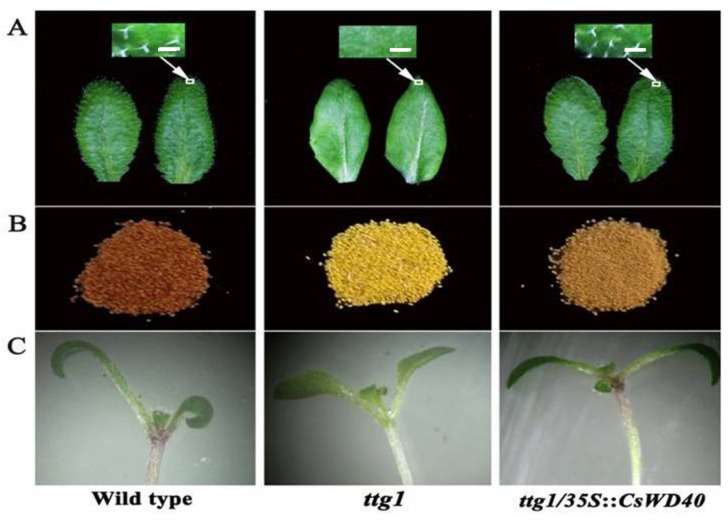
*CsWD40* complements the phenotypes of *Arabidopsis ttg1* mutant. (**A**) Leaf trichome occurrence. Bar = 0.5mm (**B**) Seed coat pigmentation. (**C**) Trichome seedling.

To determine whether CsWD40 is a functional ortholog of *TTG1* that can restore the deficient phenotypes in the *Arabidopsis ttg1* mutant, the ORF of *CsWD40*, under the control of the 35S promoter, was transformed into the mutant. The surface of the *ttg1* mutant leaves appeared smooth and there was no trichome on them. The seed coats lost the pigment store and appeared yellow in comparison with the wild type. The *ttg1* mutant lost red pigmentation in the stem of seedlings even in those induced by 6% sucrose. The results showed that overexpression of *CsWD40* fully complemented the trichome deficiency and pigmentation phenotypes in the stems of the *ttg1* mutant (Figure 2). The seed coat color of the transgenic mutant recovered partially (Figure 2).

### 2.4. Anthocyanin and PA Accumulation in the Flowers of CsWD40-Overexpressing Tobacco Plants

**Figure 3:**
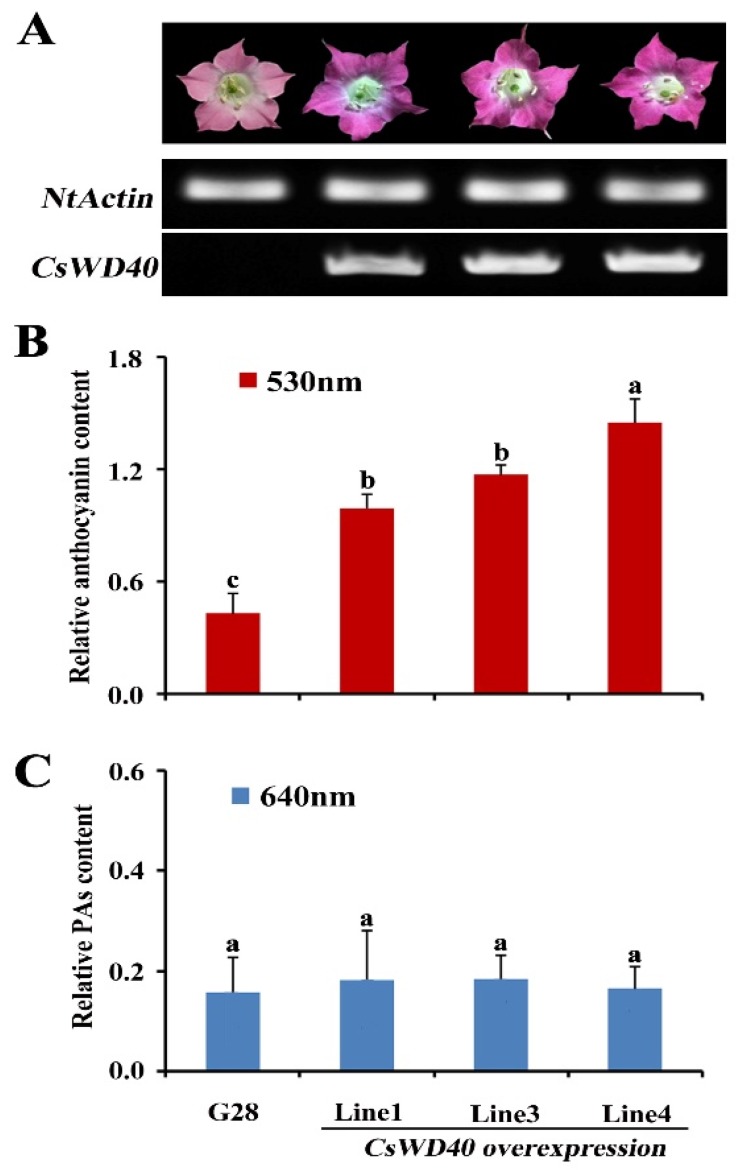
Identification of the *CsWD40* function in transgenic tobacco. (**A**) Analysis of *CsWD40* transcription levels in the flowers by semiquantitative PCR. (**B**) Relative content of anthocyanin in the flowers of *CsWD40* overexpressing tobacco. (**C**) Relative soluble proanthocyanidin content in the flowers of *CsWD40* overexpressing tobacco. All data are the means of three biological replicates, and the error bars represent the standard deviation of three replicates. Statistical significance was analyzed using ANOVA software (ANOVA ALL MAC VERSION 2.0, Thomas Hanson, OR, USA). Means followed by the same letter are not significantly different (*p* > 0.05).

To ascertain the putative function of *CsWD40*, a tobacco gene transformation system was used for ectopic expression experiments. The petals of T_1_- and T_2_-generation *CsWD40*-overexpressing tobacco plants showed a deeper pink color than those of the control (G28) (Figure 3A). The expression level of *CsWD40* was positively associated with anthocyanin content (Figure 3A,B). This result indicates that *CsWD40* is involved in anthocyanin biosynthesis. Pang reported that *MtWD40* in *Medicago truncatula* participates in PA accumulation [26]. Therefore, in this study, PA content in the flowers of transgenic tobacco plants was determined. No blue color was developed after a reaction with the DMACA (7-DIMETHYLAMINOCOUMARIN-4-ACETICACID) reagent for transgenic and wild-type tobacco plants (Figure 3C), indicating that *CsWD40* overexpression did not affect PA biosynthesis in the flowers of transgenic tobacco plants.

### 2.5. Analysis of Expression of Genes Involved in Flavonoid Biosynthesis in the Flowers of CsWD40-Overexpressing Tobacco Plants

**Figure 4:**
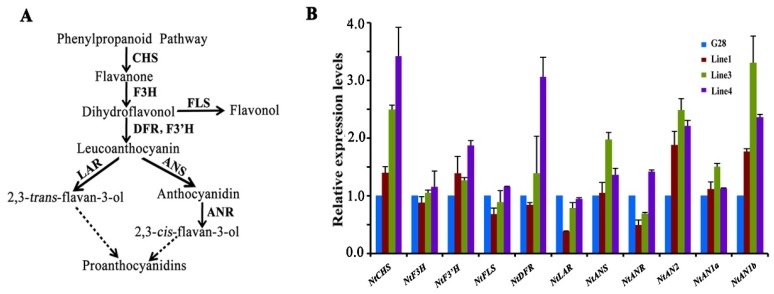
Expression of genes involved in flavonoid biosynthesis in the flowers of CsWD40-overexpressing tobacco plants. (**A**) A schematic diagram of flavonoid biosynthetic pathway. (**B**) Expression profiles of genes in flavonoid pathway in flowers of transgenic *CsWD40* tobacco lines. CHS, chalcone synthase; F3H, flavanone 3-hydroxylase; F3′H, flavonoid 3-hydroxylase; DFR, dihydroflavonol reductase; ANS, anthocyanidin synthase; ANR, anthocyanidin reductase; FLS, flavonol synthase; AN2, *N. tabacum* Anthocyanin 2; AN1a, *N. tabacum* Anthocyanin 1a; AN1b, *N. tabacum* Anthocyanin 1b.

qRT-PCR was performed to analyze the expression of genes involved in the flavonoid biosynthetic pathway in transgenic tobacco petals. The results showed that the key structural genes, *NtCHS*, *NtF3′H*, *NtDFR*, and *NtANS* were upregulated significantly (Figure 4B). Furthermore, the transcription factor genes *NtAN2* and *NtAN1b* were expressed highly in all transgenic tobacco petals (Figure 4B). *NtAN2* and *NtAN1b* were clearly activated in anthocyanin biosynthesis in tobacco [27]. These results indicate that *CsWD40* acts as a positive participator and upregulates key structural and transcript factor genes involved in the anthocyanin pathway in transgenic tobacco plants.

### 2.6. Anthocyanin and PA Content in CsWD40- and CsMYB5e-Overexpressing Tobacco Plants

The aforementioned yeast two-hybrid test results suggested that *CsWD40* could positively interact with *CsMYB5e*. To validate the synergistic effects of *CsMYB5e* and *CsWD40* function, we generated *CsWD40*- and *CsMYB5e*-overexpressing tobacco plants. We then cross-pollinated *CsWD40* and *CsMYB5e* transgenic tobacco plants in both ♀ × ♂ and ♂ × ♀ directions to produce co-overexpressing transgenic tobacco. Eleven *CsWD40*♀*CsMYB5e*♂ and nine *CsWD40*♂*CsMYB5e*♀ transgenic plants were verified by RT-PCR. There was no phenotypical difference between the flowers of *CsWD40*♀*CsMYB5e*♂ and *CsWD40*♂*CsMYB5e*♀ transgenic plants (Figure S2).

**Figure 5:**
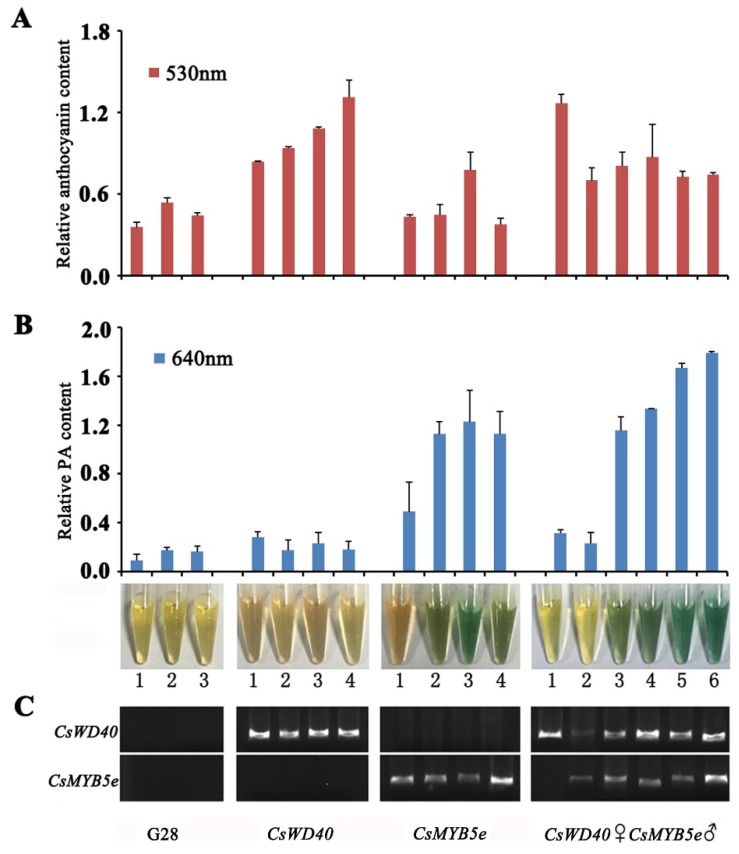
Anthocyanin and proanthocyanidin accumulation variated in petals of co-overexpressing *CsWD40* and *CsMYB5e* transgenic tobacco. (**A**) The relative content of anthocyanin was calculated on the record of absorbance at 530 nm. (**B**) The relative content of proanthocyanidin was calculated on the record of absorbance at 640 nm after reaction with DMACA regent. (**C**) RT–PCR determination of the *CsWD40* and *CsMYB5e* expression levels in *CsWD40*, *CsMYB5e*, and *CsWD40*♀*CsMYB5e*♂ transgenic tobacco flowers. All the data were present based on three biological and technical repeats.

*CsWD40* expression resulted in significantly increased anthocyanin content in the flowers of transgenic tobacco plants but did not affect PA content (Figure 5A,B). The extract solution of petals from the flowers of *CsMYB5e* lines turned blue after a reaction with DMACA (Figure 5B), indicating high PA accumulation in *CsMYB5e*-overexpressing tobacco plants, but slight changes in anthocyanin content in *CsMYB5e* tobacco plants than in control plants (Figure 5).

The data showed that anthocyanin and PA accumulation in *CsWD40*♀*CsMYB5e*♂ transgenic lines were markedly higher than those in control plants (Figure 5). In the *CsWD40*♀*CsMYB5e*♂ transgenic plants, high *CsWD40* expression and low *CsMYB5e* expression resulted in marked anthocyanin accumulation but slight PA accumulation. The highest PA accumulation was detected in lines with high *CsWD40* and *CsMYB5e* expression. The petals of *CsMYB5e* and *CsWD40* co-overexpressing transgenic tobacco plants exhibited higher levels of anthocyanin than those of lines overexpressing *CsMYB5e* alone (Figure 5, Figure S2), but anthocyanin levels were lower in the co-overexpressing tobacco plants than in the tobacco plants expressing *CsWD40* alone (Figure 5A). 

### 2.7. Expression Analysis of Genes Involved in Flavonoid Biosynthesis in CsWD40 and CsMYB5e Transgenic Tobacco

**Figure 6:**
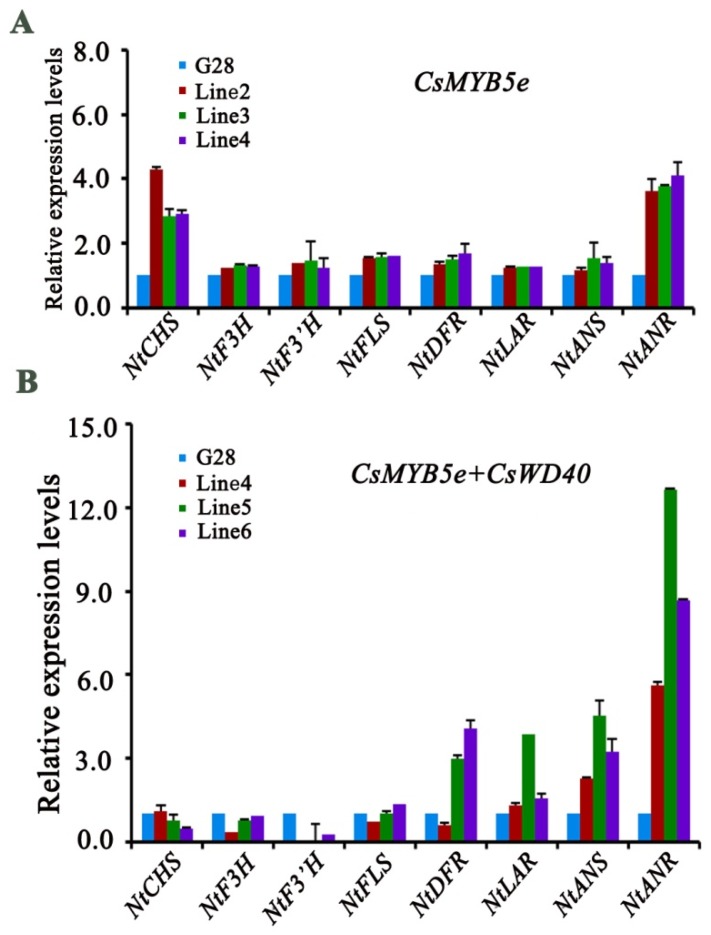
(**A**) Relative expression of flavonoid biosynthetic pathway genes in *CsMYB5e* overexpression tobacco petals. (**B**) Relative expression of flavonoid biosynthetic pathway genes in *CsWD40* and *CsMYB5e* overexpressing tobacco petals. CHS, chalcone synthase; F3H, flavanone 3-hydroxylase; F3′H, flavonoid 3-hydroxylase; DFR, dihydroflavonol reductase; ANS, anthocyanidin synthase; ANR, anthocyanidin reductase; FLS, flavonol synthase.

Gene expression profiling using qRT-PCR analysis was completed to understand the effects of overexpression of *CsWD40* and *CsMYB5e* on flavonoid pathway genes in transgenic tobacco plants. In *CsMYB5e* alone transgenic flowers, expression levels of *NtCHS* and *NtANR* increased more than two-fold (Figure 6A). In *CsWD40* (♀) and *CsMYB5e* (♂) coupled expression transgenic lines, the expression levels *NtANS* and *NtANR* increased more than four-fold and five-fold, respectively (Figure 6B). In addition, the expression levels of *NtLAR* and *NtDFR* significantly increased. The expression of other genes was either slightly increased or similar between transgenic and wild type flowers.

### 2.8. Expression Patterns of CsWD40 in Tea Leaves under Different Abiotic Stresses

**Figure 7:**
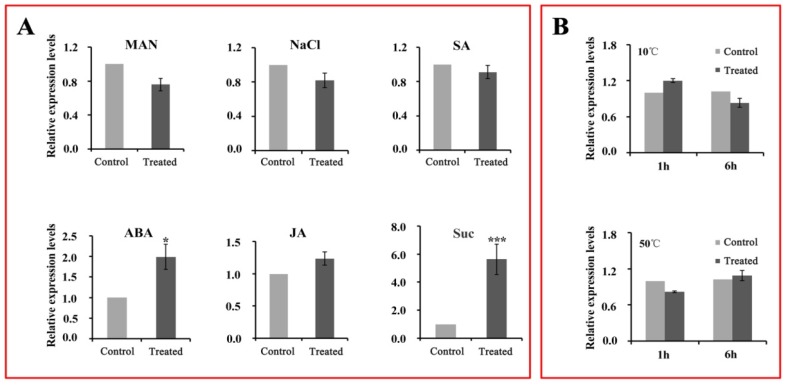
Effects of different abiotic treatments on the expression levels of *CsWD40* in tea leaves. (**A**) The transcript levels of *CsWD40* under mannitol (MAN), sodium chloride (NaCl), salicylic acid (SA), abscisic acid (ABA), jasmonic acid (JA), and sucrose (Suc) stresses. (**B**) The transcript levels of CsWD40 under different temperatures. The asterisks indicate the significant level (*n* = 3, * *p* < 0.05, *** *p* < 0.001) based on a Tukey’s honestly significant difference test.

*TaWD40D* overexpression enhanced the tolerance of *Triticum aestivum* plants to salt, mannitol, and abscisic acid (ABA) stresses, indicating that WD40 proteins may be involved in the response of plants to environmental stresses [28]. Therefore, we analyzed the expression patterns of *CsWD40* induced by different abiotic stresses, including sucrose (Suc), ABA, mannitol (Man), sodium chloride (NaCl), salicylic acid (SA), and jasmonic acid (JA) stresses. The results showed that compared to the control, the expression level of the *CsWD40* gene increased nearly 2-fold under ABA stress and approximately 6-fold under sucrose stress (Figure 7A). Under mannitol, NaCl, SA, and JA stresses, the expression level of the *CsWD40* gene varied slightly in comparison with the control (Figure 7A). To determine whether *CsWD40* expression is related to temperature changes, we compared *CsWD40* expression under low (10 °C) and high (50 °C) temperatures (Figure 7B). Our data indicated no significant difference was observed in the expression level of *CsWD40* under different temperatures.

## 3. Discussion

Flavonoid compounds are widely distributed and are ubiquitous secondary metabolites, mainly existing in the root, leaf, fruit, and skin of plants ranging from spermatophytes to mosses [9,20]. A complex comprising an R2R3-MYB TF, a bHLH domain protein, and a WD40 repeat protein regulates the production of anthocyanin and PA in seed coats, roots, leaves, and fruit [10,20,26,29]. This complex also controls the formation of root hairs and trichomes on aerial tissues in some, but not all, plants [14].

WD40 repeat proteins are widely present in plants, animals, and unicellular eukaryotes such as fungi and slime molds [30]. WD40 repeat proteins comprise a superfamily of proteins with a β-propeller structure. The core region of WD40 repeat proteins contains 40 amino acid residues, including histidine–glycine and aspartic acid–tryptophane dipeptides. The conserved motifs can be arranged in 4–16 tandems in the same protein. Eight such random repeats are present in the β-subunit of G protein in higher eukaryotic organs.

Plant flavonoid-related WD proteins are grouped into the 4 WD-repeat (4WDR) subfamily of the TTG (Transparent teata glabra) family. The functions of several WD40 repeat proteins have been reported in petunia (*Petunia hybrida*), *Perilla frutescens*, cotton (*Gossypium hirsutum*), and maize (*Zea mays*) [22,23,31]. In *A. thaliana*, only one WD40 protein (TTG1), involved in anthocyanin biosynthesis and trichome formation, has been reported [32]. The ortholog (MtWD40-1) of AtTTG1 is necessary for tissue-specific anthocyanin and PA biosynthesis in *M. truncatula* [33]. Two WD repeat genes (*GhTTG1* and *GhTTG2*) were found in *G. hirsutum* [23], but only *GhTTG1* could restore trichome formation in the *Arabidopsis ttg1* mutant and complemented the anthocyanin deficiency in the white-flowered *Matthiola incana ttg1* mutant.

A total of 195 candidate WD genes were isolated from the transcriptome data sets of *C. sinensis* [34]. However, only one *WD* gene was confirmed to be the ortholog of *AtTTG1* in tea plants. As shown in Figure 3, the petals of *CsWD40* transgenic tobacco plants accumulated a large amount of anthocyanins, but no observable PA was found in these plants. In the *Arabidopsis ttg1* mutant, *CsWD40* not only complemented trichome deficiency but also restored seed coat color. The results implied that *CsWD40* improved PA production in the seeds of the *ttg1* mutant. This result also implied the different roles of *CsWD40* in transgenic tobacco and *Arabidopsis* plants. The cause was probably the difference in the dominant flavonoid biosynthesized in the petals of tobacco plants and the seeds of *Arabidopsis* plants. Anthocyanin is mainly biosynthesized in the petals of tobacco plants [35], whereas PA is mainly biosynthesized in the seeds of *Arabidopsis* plants [17].

Besides, not only the structural genes, *NtCHS*, *NtF3*′*H*, *NtDFR*, and *NtANS*, but also the transcript factor genes, *NtAN2* and *NtANb1*, were upregulated significantly in *CsWD40* transgenic tobacco petals (Figure 5B). A potential reason for this is that CsWD40 improves the stability of some MBW complexes or promotes the formation of some MBW complexes. These complexes may regulate the gene expression of some transcription factors. For example, TT8 expression was found to be directly regulated by TT8 itself through a positive feedback regulatory loop involving redundant MBW complexes [36,37]. TT8 promoter activity is itself was partially regulated by TT1 [37].

The anthocyanin and PA biosynthetic pathways are regulated by different MBW complexes. Different MYB TFs play critical roles in the regulation of the complexes [24]. For example, TT2 (ATMYB123) in subgroup 5 mainly regulates PA biosynthesis in *Arabidopsis*. PAP1 (ATMYB75), in subgroup 6, significantly promotes anthocyanin accumulation in *Arabidopsis* [38]. In our study, CsWD40 could interact with CsAN2 (the ortholog of PAP1) and CsMYB5e (the ortholog of TT2) in the yeast two-hybrid system (Figure 1). In our previous study, *CsAN2* markedly increased anthocyanin biosynthesis in *CsAN2*-overexpressing tobacco plants [39]. In *CsMYB5e* transgenic tobacco plants, the PA pathway was markedly upregulated [40]. Expression levels of both anthocyanin and PA biosynthesis related genes were upregulated to varying degrees in the petals of *CsWD40* and *CsMYB5e* co-overexpressing transgenic tobacco plants. *CsWD40* and *CsMYB5e* co-overexpressing transgenic tobacco plants tended to show less anthocyanin production than transgenic tobacco plants expressing *CsWD40* alone. This might be because *CsWD40* promoted the regulation effects of *CsMYB5e*, resulting in higher PA biosynthesis than anthocyan in biosynthesis. In our previous studies, *CsANR* overexpression reduced anthocyanin production and promoted PA accumulation in the petals of tobacco plants [41]. In the *Arabidopsis* mutant of *BAN* (loss of function of *ANR*), a high level of anthocyanin accumulated in the seed coat. Hybridization and yeast two-hybrid assays further confirmed that CsWD40 played a crucial role in the regulation of anthocyanin and PA biosynthesis through the formation of MBW ternary complexes [42].

In a previous study, the ectopic overexpression of *TaWD40D* (*T. aestivum* L.) in *Arabidopsis* greatly increased the tolerance of the plants to ABA (Abscisic acid), salt, and osmotic stresses during seed germination and seedling development [28]. In this study, the transcript levels of the *CsWD40* gene were significantly upregulated under sucrose and ABA stresses, but no response was found to mannitol, NaCl, and SA (Salicylicacid) stresses under different temperatures. Our previous study showed that under ABA and sucrose stresses the most flavonoid was accumulated and the related gene expression increased [43]. This result suggests *CsWD40* promotes the accumulation of products of the flavonoid biosynthetic pathway, which led to stress tolerance in tea plants.

## 4. Materials and Methods

### 4.1. Plant Materials and Growth Conditions

Numerous samples from different organs (including young sprout, young stem, and tender root) of the tea plants growing on the grounds of the research station at Anhui Agricultural University were obtained during the growth period in early spring. All samples were immediately frozen in liquid nitrogen and were stored at −80 °C for the present investigation.

Wild-type tobacco “G28” plants were used in genetic transformation studies. Tobacco plants were grown in a controlled environment chamber at a constant temperature of 28 ± 3 °C and a 12/12-h (light/dark) photoperiod with a light intensity of 150–200 μmol·m^–2^·s^–1^.

*A. thaliana* (Ecotype Columbia) *ttg1* mutant seeds were purchased from the *Arabidopsis* Seed Bank (http://www.Arabidopsis.org/). The methods used for seed germination and plant growth for *A. thaliana* were the same as those used for tobacco [44]. For genetic transformation, *A. thaliana* plants were grown in a green house. The temperature, light intensity, and photoperiod were 22 ± 2 °C, 50 µmol·m^−2^·s^−1^, and 16/8 h (light/dark), respectively.

In order to induce the anthocyanin accumulation in seedlings of *Arabidopsis thaliana*, *Arabidopsis* seeds were surface sterilized using 70% alcohol for 1 min followed by 3 times of washing with sterile distilled water, then treated with 1.5% sodium hypochlorite (10% Clorox), (SC Johnson Wax, Racine County, WI, USA) for 8 min followed by 6 times of washing with sterile distilled water. Seeds were germinated on MS medium with 6% sucrose. Seedlings were cultured for five days after germination and their phenotypes were observed under microscope.

### 4.2. Cloning CsWD40 and CsMYB5e

Total RNA was isolated from plants using the RNAiso-mate for Plant Tissue Kit (Takara, Dalian, China), according to the manufacturer’s protocol. cDNAs were reverse transcribed using the PrimeScript^®^ RT Reagent Kit (Takara, Dalian, China), according to the manufacturer’s protocol. The specific conditions of PCR were as follows: 98 °C for 30 s; followed by 30 cycles of 98 °C for 10 s and 60 °C for 20 s; 72 °C for 30 s; and then a 10-min extension step at 72 °C. The cDNAs of *CsGL3*, *CsTT8*, *CsAN2*, and *CsMYB5e* were provided by Tong Li and Xiaolan Jiang in our university.

### 4.3. Extraction and Analysis of Anthocyanins and PAs

To extract anthocyanins and PAs, the samples were ground in liquid nitrogen and extracted with extraction solution (0.5% HCl:80% methanol:19.5% water) by vortexing and then sonicating for 30 min at low temperature (4 °C). The samples were centrifuged at 5000× *g* for 20 min, and the residues were re-extracted twice. The supernatants were combined and diluted with extraction solution to 2 mL. Absorbance of the aqueous phase was measured at 530 nm. The extraction method for PA is described in the article by Jiang [45].

The total PA content was determined spectrophotometrically at 640 nm after a reaction with the DMACA reagent (0.2% (*w*/*v*) DMACA in methanol-3 N HCl), with (−)-epicatechin serving as the standard [33].

### 4.4. Agrobacterium-Mediated Transformation of Arabidopsis and Tobacco

The ORFs of *CsWD40* were cloned into a binary vector pDONR207 by using the Gateway^®^ Cloning System (Invitrogen, Carlsbad, CA, USA). The primers used are listed in Table S1. The vectors were constructed and the genetic transformation of tobacco was performed according to the method of Li et al. [43]. The phenotypes of transgenic plants growing in the controlled environment chamber were recorded to characterize the effect of transgene overexpression on growth.

### 4.5. Hybridization of Different Transgenic Tobacco Lines

The T_1_-generation plants of *CsWD40* and *CsMYB5e* transgenic tobacco plants were chosen for research. Transgenic tobacco plants separately overexpressing *CsMYB5e* and *CsWD40* were crossed in both ♀ × ♂ and ♂ × ♀ directions. The pollinated ovaries were collected until the seeds ripened. The next generation of plants was grown for subsequent experiments after PCR verification.

### 4.6. Abiotic Stress Treatment of Tea Shoots

For abiotic stress treatment, tea shoots sprouting approximately 10 cm were cultivated in 90 mM sucrose, 200 mM mannitol, 50 mM NaCl, 10 µM JA, 100 µM ABA, and 20 mM SA for 12 h. All samples were exposed to treatments at 22 °C, with a light intensity of 150–200 µmol·m^−2^·s^−1^. Control shoots were cultivated in deionized water. For low- and high-temperature treatment, the shoots were exposed to treatments at 10 and 50 °C for 1 and 6 h, respectively.

### 4.7. Yeast Two-Hybrid Assay

The plasmids pGADT7 (Clontech Laboratories, Inc., Terra Bella Ave, Mountain View, CA, USA) and pGBKT7 (Clontech Laboratories, Inc., Terra Bella Ave, Mountain View, CA, USA), which contained the GAL4 activation domain and the GAL4 DNA-binding domain, were used for yeast two-hybrid experiments.

To analyze protein interaction, the ORFs of *CsWD40*, *CsGL3*, *CsTT8*, *CsAN2*, and *CsMYB5e* were cloned into the pGADT7 (Clontech Laboratories, Inc., Terra Bella Ave, Mountain View, CA, USA) and pGBKT7 vectors through pfu DNA PCR (Thermo Scientific, Waltham, MA, USA). This cloning was performed using the Matchmaker™ Gold Yeast Two-Hybrid System (Clontech), according to the manufacturer’s instructions described in Clontech Yeast Protocol Handbook. The primers with leading and tailing EcoRI and BamHI restriction enzyme sites were used and are listed in Table S1.

BD-*CsWD40*, *CsGL3*, *CsTT8*, *CsAN2*, or *CsMYB5e* and AD-*CsWD40*, *CsGL3*, *CsTT8*, *CsAN2*, or *CsMYB5e* were cotransformed into the yeast strain Y2HGold (Clontech) using the PEG/LiAC method described in the Clontech Yeast Protocol Handbook. These transformed colonies were tested on synthetic dropout (SD) medium with X-α-Gal lacking leucine, tryptophan, histidine, and adenine (SD/-Ade/-His/-Leu/-Trp).

### 4.8. Quantitative Real-Time PCR

RNA was extracted from various tissues and was quantified spectrophotometrically (NANODROP 2000, Thermo Scientific). Reverse transcription of RNA into cDNA was performed using 2 μL of 5× PrimeScript RT Master Mix (Takara) and 500 ng of RNA in a reaction volume of 10 µL.

In addition, cDNA was diluted to 25% (*v*/*v*) with deionized water before being used as the template. Quantitative real-time PCR was performed in a reaction volume of 20 µL containing 10 µL of SYBR Green PCR Master Mix (Takara), 1.1 µL of cDNA, and 0.8 µL of forward and reverse primers (10 μM). The PCR cycling parameters used were as follows: 95 °C for 30 s and 40 cycles of 95 °C for 5 s, 30 s at 60 °C, and 30 s at 72 °C, followed by melting curve analysis from 55 to 95 °C. The transcription abundance was normalized to the transcription abundance of the control gene and was calculated from three technical replicates. The gene *glyceraldehyde-3-phosphate dehydrogenase* (*GAPDH*, Accession No. GE651107) in tea plants and *ribosomal protein L25* (*RPL25*, Accession No. L18908) in tobacco were used as control genes in qPCR analysis. Their gene primers were listed in Table S1. The relative expression level was calculated using a previously described method [46].

## Supplementary Materials

Supplementary materials can be found at http://www.mdpi.com/1422-0067/19/6/1686/s1.

## Abbreviations

ANR	Anthocyanidinreductase
ANS	Anthocyanidin synthase
CHS	Chalcone synthase
DFR	Dihydroflavonolreductase
DMACA	Dimethylaminocinnamaldehyde
F3H	Flavanone 3-hydroxylase
F3′H	Flavonoid 3-hydroxylase
FLS	Flavonol synthase
MBW	MYB-bHLH-WD40
Pas	Proanthocyanidins
ttg1	Transparent testa glabra 1
