# Supplementary material for: A WD40 Repeat Protein from Camellia sinensis Regulates Anthocyanin and Proanthocyanidin Accumulation through the Formation of MYB–bHLH–WD40 Ternary Complexes
Source: Int J Mol Sci. 2018 Jun 6;19(6):1686. doi: 10.3390/ijms19061686 (PMC6032167; doi:10.3390/ijms19061686)
Supplement: Supplementary file 1 [file ijms-19-01686-s001.pdf]

A WD40 Repeat Protein from Camellia sinensis Regulates Anthocyanin and Proanthocyanidin Accumulation Through the Formation of MYB–bHLH–WD40 Ternary Complexes

Yajun Liu[1,†], Hua Hou[1,†], Xiaolan Jiang[2,†], Peiqiang Wang[2], Xinlong Dai[2], Wei Chen[2], Liping Gao[1*], and Tao Xia[2*]

TableS 1. The specific primers used in this study.

purpose	Gene names	primer name	primer sequence (5'-3')
cloning	*CsWD40*	*CsWD4*0-attB-HA-F	GGGGACAAGTTTGTACAAAAAAGCAGGCTATGTACCCATACGATGTTCCAGATTACGCTATGGAGAATTCGAGCCAAG
		CsWD40-attB-HA-R	GGGGACCACTTTGTACAAGAAAGCTGGGTTCAAACTTTCAGAAGCTGC
		End-to-end F	ATGGAGAATTCGAGCCAAG
		End-to-end R	GATCTCAAACTTTCAGAAGCTGC
Real time PCR	*CsWD40*	qRT-PCR-F	AAACTCCGTGACCTACGACTCCCCG
		qRT-PCR-R	CTTGGTGGGAGGGTAAGGGTGTTCG
Construction of protein expression vectors	*CsWD40*	*CsWD40*-AD-F	ATGGAGGCCAGTGAATTCATGGAGAATTCGAGCCAAG
		CsWD40-AD-R	CTCGAGCTCGATGGATCCTCAAACTTTCAGAAGCTGC
		CsWD40-BD-F	GCCATGGAGGCCGAATTCATGGAGAATTCGAGCCAAG
		CsWD40-BD-R	CTGCAGGTCGACGGATCCTCAAACTTTCAGAAGCTGC
	CsMYB5a	*CsMYB5a*-AD-F	ATGGAGGCCAGTGAATTCATGGGGAGGAGTCCATGCTG
		CsMYB5a-AD-R	CTCGAGCTCGATGGATCCTCATGGCCAGTCCTCAGAAT
		CsMYB5a-BD-F	GCCATGGAGGCCGAATTCATGGGGAGGAGTCCATGCTG
		CsMYB5a-BD-R	CTGCAGGTCGACGGATCCTCATGGCCAGTCCTCAGAAT
	CsMYB5e	*CsMYB5e*-AD-F	ATGGAGGCCAGTGAATTCATGGGAAGGGCTCCTTGTTG
		CsMYB5e-AD-R	CTCGAGCTCGATGGATCCTCAGATCAACAAAGATTCAG
		CsMYB5e-BD-F	GCCATGGAGGCCGAATTCATGGGAAGGGCTCCTTGTTG
		CsMYB5e-BD-R	CTGCAGGTCGACGGATCCTCAGATCAACAAAGATTCAG
	CsAN2	*CsAN2*-AD-F	ATGGAGGCCAGTGAATTCATGGACATTGTTTGTTGTGTTC
		CsAN2-AD-R	CTGCAGGTCGACGGATCCTCATTCATCACCTAACAGATCC
		CsAN2-BD-F	GCCATGGAGGCCGAATTCATGGACATTGTTTGTTGTGTTC
		CsAN2-BD-R	CTGCAGGTCGACGGATCCTCATTCATCACCTAACAGATCC
	CsTT8	*CsTT8*-AD-F	ATGGAGGCCAGTGAATTCTCCTGGTGTTGGATTACC
		Cs TT8-AD-R	CTCGAGCTCGATGGATCCTCAGTTCTGGGGTATTAT
		Cs TT8-BD-F	GCCATGGAGGCCGAATTCTCCTGGTGTTGGATTACC

		Cs TT8-BD-R	CTGCAGGTCGACGGATCCTCAGTTCTGGGGTATTAT
	*Cs*GL3	*CsGL3*-AD-F	ATGGAGGCCAGTGAATTCATGTGTTGGGCAATGGCT
		CsGL3-AD-R	CTCGAGCTCGATGGATCCCTAACACTTGCCAGCAAT
		CsGL3-BD-F	GCCATGGAGGCCGAATTCATGTGTTGGGCAATGGCT
		CsGL3-BD-R	CTGCAGGTCGACGGATCCCTAACACTTGCCAGCAAT
Quantitative RT-PCR	NtCHS	*NtCHS*-qRT- F	TGCTAAGCGAATACGGGAAC
		NtCHS-qRT- R	AACAGAAACACTGCGGAGGA
	NtF3'H	*NtF3'H*-qRT- F	TTGTCCCGCAATGACTTACG
		NtF3'H -qRT- R	GCATAGTAGGTAGGCGAGGTG
	NtDFR	*NtDFR*-qRT- F	GCGAAAGGGAGGTATATGTGCTC
		NtDFR -qRT- R	TGCTTGTCCCTCGGTACTCAGTA
	NtFLS	*NtFLS*-qRT- F	TGAAGGGAAAAGGGGTTGG
		NtFLS-qRT- R	ACTCCTCATTTGCTTCCCTGTAG
	NtLAR	*NtLAR*-qRT- F	GCAGCAGAAGACTATAGAACTGTG
		NtLAR -qRT- R	CATGTGTTAGAGCTGCAACTACAC
	NtANS	*NtANS*-qRT- F	ACTACTACCCCAAATGTCCCCAAC
		NtANS -qRT- R	CCGTTACCCACTGTCCTTCATAGA
	NtANR	*NtANR*-qRT- F	CTTGAAGGGTATGCAGATGTT
		NtANR-qRT- R	GCAGAGCAAACATATCGTCCAG
	NtAN2	*NtAN2*-qRT- F	GAAGAAAGGTGCATGGACTG
		NtAN2-qRT- R	TCTGCAGCTCTTTCTGCATC
	NtAN1a	*NtAN1a*-qRT- F	ACCATTCTCGAACACCGAAG
		NtAN1a-qRT- R	TGCTAGGGCACAATGTGAAG
	NtAN1b	*NtAN1b*-qRT- F	CTTGAACACTTCTCA AACCGA
		NtAN1b-qRT- R	TGCTAGGGCACAATGTGAAG

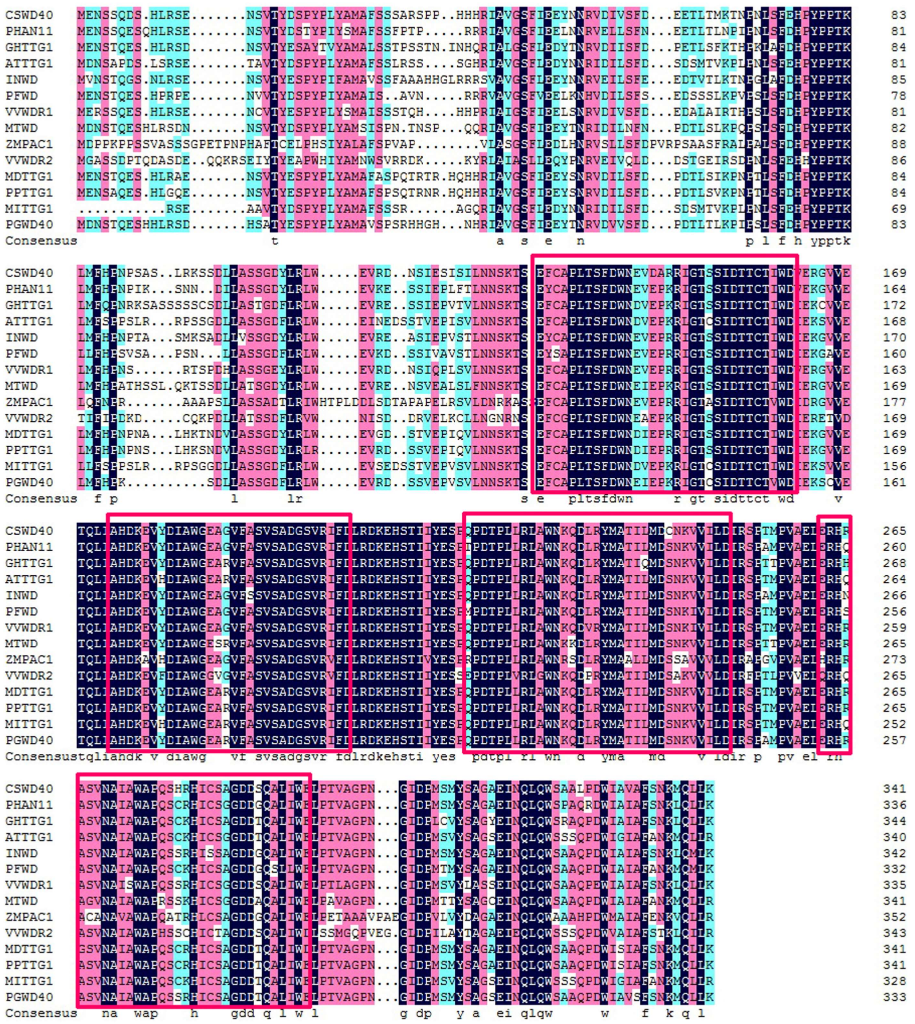

Figure S1. Amino acid alignment of *Cs*WD40 proteins from tea plants and its orthologs from other plants

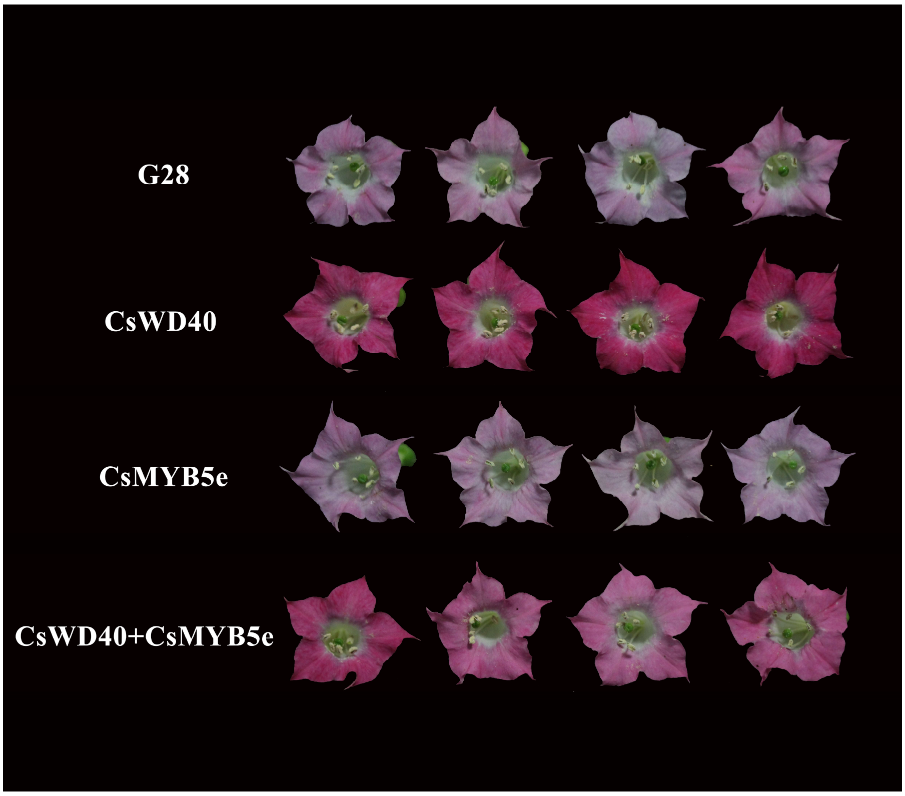

Figure S2. Flower pigmentations of *CsWD40*, *CsMYB5e*, and *CsWD40*♀*CsMYB5e*♂ (or *CsWD40*♂*CsMYB5e*♀) transgenic tobaccos.
